# Structure Solution of Nano-Crystalline Small Molecules Using MicroED and Solid-State NMR Dipolar-Based Experiments

**DOI:** 10.3390/molecules26154652

**Published:** 2021-07-31

**Authors:** Nghia Tuan Duong, Yoshitaka Aoyama, Katsumi Kawamoto, Toshio Yamazaki, Yusuke Nishiyama

**Affiliations:** 1 RIKEN-JEOL Collaboration Center, RIKEN, Yokohama, Kanagawa 230-0045, Japan; nghiatuan.duong@riken.jp; 2 JEOL Ltd., Akishima, Tokyo 196-8558, Japan; yaoyama@jeol.co.jp; 3 System in Frontier Inc., Tachikawa, Tokyo 190-0012, Japan; KatsumiKawamoto@sifi.co.jp; 4 RIKEN Center for Biosystems Dynamics Research, RIKEN, Yokohama, Kanagawa 230-0045, Japan; toshio.yamazaki@riken.jp; 5 JEOL RESONANCE Inc., Akishima, Tokyo 196-8558, Japan

**Keywords:** microED, NMR crystallography, GIPAW calculations, ^1^H-^14^N PM-S-RESPDOR, ^1^H-^1^H SERP

## Abstract

Three-dimensional electron diffraction crystallography (microED) can solve structures of sub-micrometer crystals, which are too small for single crystal X-ray crystallography. However, R factors for the microED-based structures are generally high because of dynamic scattering. That means R factor may not be reliable provided that kinetic analysis is used. Consequently, there remains ambiguity to locate hydrogens and to assign nuclei with close atomic numbers, like carbon, nitrogen, and oxygen. Herein, we employed microED and ssNMR dipolar-based experiments together with spin dynamics numerical simulations. The NMR dipolar-based experiments were ^1^H-^14^N phase-modulated rotational-echo saturation-pulse double-resonance (PM-S-RESPDOR) and ^1^H-^1^H selective recoupling of proton (SERP) experiments. The former examined the dephasing effect of a specific ^1^H resonance under multiple ^1^H-^14^N dipolar couplings. The latter examined the selective polarization transfer between a ^1^H-^1^H pair. The structure was solved by microED and then validated by evaluating the agreement between experimental and calculated dipolar-based NMR results. As the measurements were performed on ^1^H and ^14^N, the method can be employed for natural abundance samples. Furthermore, the whole validation procedure was conducted at 293 K unlike widely used chemical shift calculation at 0 K using the GIPAW method. This combined method was demonstrated on monoclinic l-histidine.

## 1. Introduction

X-ray diffraction (XRD) crystallography is a fundanmental technique for structural determination in chemistry and biology because it provides three-dimensional structure at the atomic level. However, this technique requires that the target molecule must yield sufficiently large crystals. Such a requirement precludes the applications of XRD crystallography to micro- or nanocrystals. Powder XRD (PXRD) is a potential solution; however, it suffers from the ambiguities in (i) localizing hydrogen atoms and (ii) distinguishing the atoms with similar atomic numbers such as carbon, nitrogen, and oxygen not to mention the requirement of a large sample amount of isomorphic powder crystals (about 1 mg). Recently, microED, three-dimensional electron diffraction crystallography, has gained attraction since it is able to solve the crystalline structure from submicron-sized single crystals including small molecules to proteins [1,2,3,4,5,6,7,8,9,10,11,12,13]. This advantage is owing to stronger interactions of the electron beam to matter than the X-ray beam, thus allowing the studies of crystals smaller than 1 μm. In crystallography, R factor, which gives agreement between experimental diffraction data and modeled structure, is typically used to validate the structures. However, structures solved by microED with the kinetic analysis give a relatively high (between 15–30% for small molecules) R factors, that are well above the satisfactory level in XRD (<7%). Such a high value is largely due to dynamic scattering which redistributes the strong diffraction intensities to weak spots. As a result, small R factor may not necessarily represent better structure or R factor may not be a reliable measure of validation as long as kinetic analysis is used. Indeed, microED struggles to (i) precisely locate hydrogen (H) atom due to its low scattering and (ii) unambiguously assign carbon (C), nitrogen (N), and oxygen (O) atoms due to their similar atomic numbers, while (P)XRD partially shares the same issues. The inclusion of dynamic refinement in the data analysis significantly reduces the R factor and can address the above issues, even determining the absolute configuration [7,14,15,16]. However, size, thickness and orientation of the crystals have to be involved in strict treatments. Nevertheless, these two limitations make the complete structure determination solely by microED difficult, thus necessiating the use of additional techniques.

We have recently demonstrated that solid-state nuclear magnetic resonance (ssNMR) can provide complementry information to microED-based structures, allowing efficient validation of structures [4]. Namely, the limitations posed by microED can be readily solved by ssNMR since (i) ^1^H is the most sensitive NMR attive nucleus due to its high gyromagnetic ratio and natural abundance; whereas (ii) ^13^C, ^14/15^N, and ^17^O NMR are completely different nuclei due to their intrinsic characteristics—gyromagnetic ratios, spin numbers, and natural abundances. Indeed, ssNMR has intensively been used to solve similar issues for XRD-based structures, namely poor locations of hydrogen atoms and ambiguous assignments of isoelectronic moeities like F, OH, and CH_3_, etc. It has been demonstrated that ssNMR, diffraction techniques (mostly XRD), and quantum chemical calculation can be combined for structure determination, an approach referred to as NMR crystallography [17,18,19,20,21,22,23,24]. A common practice of NMR crystallography utilizes the NMR chemical shifts as a measure of validation instead of R factor. Although chemical shifts are sensitive to the local environments surrounding a nucleus, these values cannot be directly converted to the crystallographic structure. Thus, to link the chemical shift and the structure, a quantum chemical calculation, such as GIPAW (gauge including projector argumented wave) method [25,26,27], is desired since it can optimize the structure and estimate the shieldings, thus chemical shifts from the structure. The strategy is that once candidate structures have been modeled by a diffraction method, computation, and their combination, these caculated chemical shifts are then compared to experimental NMR values to validate the correct structure. While most of the application of NMR crystallography use XRD-based structure, we have successfully applied the NMR crystallography approach using ssNMR and microED to determine crystalline structure including hydrogens, enabling structural solution from nano-sized crystals of orthorhombic l-histidine and the B form of cimetidine [4].

The successful application of NMR crystallography relies on the precise estimate of chemical shift values. Although GIPAW calculations yield root mean square deviation (RMSD) of 0.3–0.6 ppm for ^1^H and 2–3 ppm for ^13^C in most cases, several examples show poor agreement [28,29,30,31]. This discrepancy results from the limited computational level and lack of molecular motions in the calculations. Indeed, GIPAW calcualtions are performed at 0 K, whereas NMR experiments are typically conducted at 293 K or ambient temperature. Such a difference could lead to wrong estimate of strength of hydrogen bonding, since proton position can be labile to temperature [32]. The temperature effect can be involved in chemical shift calculation using density functional theory (DFT)-molecular dynamics [33]. 

Here, we propose NMR dipolar-based as an alternative or additive method to validate the structures instead of or in addition to chemical shift and R factor. Unlike chemical shift, dipolar coupling is directly related to structure because dipolar coupling is inversely proportional to the cube of distance, thus sensitive to the arrangements of atoms within a molecule. Although the spin dynamics are complicated when multiple dipolar couplings are involved, the NMR dipolar-based data can be simulated straightforwardly for a given structure using spin dynamics numerical simulations. This reminds us the role of calculated chemical shift; hence, NMR dipolar-based data can also be used in the NMR crystallography. The structure solution is fulfilled by evaluating the degree of agreement between experimental and simulated NMR dipolar-based data. The NMR dipolar-based data can be used solely or in combination with a quantum computation to refine the microED structures, thus improving the accuracy. As all the NMR measurements are performed at the ambient temperature, the temperature-dependent effect issue can be lifted. In this work, we use the combination of microED and ssNMR dipolar-based experiments together with spin dynamics numerical simulations to determine the structure of moloclinic l-histidine (His). It is worth noting that the l-histidine sample exhibits two stable crystalline forms: monoclinic and orthorhombic. In a recent work from our lab, the orthorhombic l-histidine was used as a model for de-novo structural determination by the combination of microED, ssNMR experiments, and GIPAW calculation [4]. Herein, we choose monoclinic l-histidine as the model sample for a proof of concept. The two NMR dipolar-based experiments, namely ^1^H-^14^N rotational-echo saturation-pulse double-resonance (RESPDOR) [34,35,36,37,38,39] and ^1^H-^1^H selective recoupling of proton (SERP) [40,41,42], were used. Both experiments were performed at fast magic-angle spinning (>60 kHz) for proton-detection and a high-resolution ^1^H spectrum. The structure of His was firstly analyzed by microED to yield candidate structures whose correctness needs to be identified. By performing ^1^H-^14^N RESPDOR experiment, we unambiguosly assigned the C and N atoms; whereas, by performing ^1^H-^1^H SERP, we clearly verified the position of the H atom on which N atoms on the imidazole ring. The structural information gained by ssNMR dipolar-based experiments helps complete the structural determination by microED. We also perform the quantum chemical calculation to verify the final structure.

## 2. Experiments and Methods

### 2.1. Sample

l-histidine powder (Wako Pure Chemical Industries Ltd., Japan) was dissolved in ethanol/water solution (about 40 *w/w*%) at 343 K. Monoclinic form of l-histidine was recrystalized by slow evaporation and was separated by filtration. The recrystallized sample was dried at 313 K and identified by ^1^H DQ/^1^H SQ solid-state NMR at a MAS frequency (*ν*_R_) of 70 kHz [43].

### 2.2. Three-Dimensional Electron Diffraction Crystallography (MicroED)

The ED patterns of the monoclinic l-histidine crystals were measured using a JEM-F200 transmission electron microscope (JEOL Ltd., Tokyo, Japan) operating at 200 kV. The sample was maintained at the ambient temperature during measurements. The diffraction data were recorded using a high-sensitivity CMOS camera (TemCam XF-416, TVIPS GmbH, Gauting, Germany) with × 4 binning (1024 × 1024 pixels). The camera length (874 mm) was calibrated using a gold polycrystal specimen. In order to minimize the dose damage, electron dose rate was set to a very low level of 0.01 elÅ^−2^s^−1^. A set of ED patterns was collected under the continuous rotation from −30° to +30° with a rate of 0.25° s^−1^ using RECORDER software (System In Frontier Inc., Tokyo, Japan). This condition yielded a total irradiation time of 240 s, corresponding to 2.4 elÅ^−2^ total dose. Exposure time was set to 4 s, giving 60 images in total. The granular particles of monoclinic l-histidine crystals were grounded by glass plate and distributed on a TEM grid supported by ultra-thin carbon film. The collected ED patterns were indexed and converted into the SHELX format using XDS [44]. Initial structure was solved by SIR2019 using direct method [45]. The solutions were refined after correcting atomic assignments and adding protons by SHELXL program with ShelXle interface [46,47]. 

### 2.3. Solid-State Nuclear Magnetic Resonance (NMR)

All NMR experiments were recorded at ambient temperature on either 600 MHz (JNM-ECZ600R, JEOL RESONANCE Inc., Tokyo, Japan) or 700 MHz (JNM-ECZ700R, JEOL RESONANCE Inc., Tokyo, Japan) spectrometers equipped with 1.0 mm ^1^H/X double-resonance ultrafast MAS probes. The sample was packed into a 1.0 mm zirconia rotor. The following three experiments were performed at the 700 MHz spectrometer at *ν*_R_ of 70 kHz and the recycling delay (RD) of 10.0 s, unless otherwise stated. The SERP experiment was performed at the 600 MHz spectrometer at *ν*_R_ of 68.03 kHz and the RD of 8.5 s.

#### 2.3.1. ^1^H Double-Quantum (DQ)/^1^H Single-Quantum (SQ) Correlation

A $R18_{4}^{7}\;$DQ homonulcear recoupling sequence [48] as shown in Figure S1a was used to excite and reconvert the ^1^H DQ coherences in the ^1^H DQ/SQ experiments. The ^1^H radio-frequency (rf) field (ν_1H_) was set to 158 kHz for the $R18_{4}^{7}\;$sequence and 281 kHz for a read-pulse of 0.89 µs. The $R18_{4}^{7}\;$ mixing time was 114.2 µs. Before the read-pulse, the 0.5 ms z-filter was employed to suppress residual transversal magnetization. The two-dimensional (2D) ^1^H DQ/SQ spectrum was recorded with 4 scans, 64 *t*_1_ points, and rotor-synchronized *t*_1_ increment of 57.1 µs. The experimental time was 1.4 h. The States-TPPI method was employed for the quadrature detection in the indirect dimension [49].

#### 2.3.2. ^1^H-{^13^C} Proton-Detected CP-HSQC 

The experiments were performed using the sequence in Figure S1b. The ν_1H_ and ν_13C_ for π/2 pulse were 281 kHz and 220 kHz, respectively. The ^1^H → ^13^C and ^13^C → ^1^H cross-polarization (CP1 and CP2, respectively) were performed using ν_1H_ = 20 kHz and ν_13C_ = 50 kHz with a linear ramp on the ^13^C channel of −14% for CP1 and +14% for CP2. The contact time of CP1 was 2.0 ms while that of CP2 was either 0.1 ms or 1.0 ms. The 100 ms HORROR scheme with ν_1H_ = 35 kHz was used to suppress the residual ^1^H polarizations after CP1. The WALTZ decoupling sequences, with ν_1H_ = ν_13C_ = 10 kHz, were used during the ^13^C evolution period and the ^1^H acquisition, respectively. Each 2D ^1^H-{^13^C} spectrum was recorded with 24 scans, 128*t*_1_ points on the ^13^C indirect spectral width of 180 ppm. The experimental time was 17.1 h. The States-TPPI method was employed for the quadrature detection in the indirect dimension [49].

#### 2.3.3. ^1^H-^14^N Phase-Modulated Rotational-Echo Saturation-Pulse Double-Resonance (PM-S-RESPDOR)

The *ν*_R_ was reduced to 62.5 kHz for spinning stability. The experiment was performed using the sequence in Figure S1c. The ^1^H pulse lengths for π/2 and π pulses were 0.93 and 2.13 µs, respectively. The $\mathrm{SR}4_{1}^{2}\;$sequence was used to recouple ^1^H-^14^N dipolar couplings [50]. The ν_1H_ value for the $\mathrm{SR}4_{1}^{2}\;$sequence was 125 kHz. The *τ*_PM_ and ν_14N_ for the phased-modulated (PM) pulse [38,51,52,53,54] were 0.192 ms (= 12 rotor cycles) and 140 kHz, respectively. The ^1^H and ^14^N offsets were 7 ppm and 0 ppm, respectively. The mixing time was ranged from 0 to 3456 µs with a step of 64 µs. The NMR measurement was recorded with 18 scans and the RD was 6 s. The experimental time was 3.3 h.

#### 2.3.4. ^1^H-^1^H Selective Recoupling of Proton (SERP)

The experiments were performed using the sequence in Figure S1d. The shaped Q3 Gaussian pulse was applied at 14.28 ppm to select imidazole N**H** with the pulse length and rf-field of 2.0 ms and 1.8 kHz, respectively. The selective N**H** to H6 and N**H** to H8 SERP build-up curves were performed at ^1^H offsets of 9.73 ppm and 10.60 ppm, respectively. The cycle time, *ν*_1H_, phase, and *τ*_mixing_ for SERP for both experiments were 14.7 µs, 108 kHz, 480°, and from 0 to 3175.2 µs with a step of 58.8 µs, respectively. The ^1^H pulse length for the read-pulse was 0.75 µs. Both experiments were recorded with 24 scans with the recycling delay of 8.5 s, resulting in the experimental time of 3.2 h.

### 2.4. Spin Dynamics Simulation

Numerical simulations were performed using the SIMPSON software [55,56,57]. The input files are available in Supplementary Materials. For the ^1^H-^14^N PM-S-RESPDOR experiment, the powder averaging was performed with 100 pairs of {α_PR_, β_PR_} angles according to the REPULSION algorithm and 11 γ_PR_ angles. The spin models consist of a specific H spin and its neighboring N spins within the distance of 4.0 Å, corresponding to the four candidate structures of His1-4. For all the PM-S-RESPDOR simulations, the ^14^N quadrupolar coupling constant was set to 1.0 MHz. The *J*-couplings and the anisotropic chemical shifts of ^1^H and ^14^N nuclei were neglected. The other conditions were identical to the experiment. For the ^1^H-^1^H SERP experiments, the powder averaging was performed with 143 pairs of {α_PR_, β_PR_} angles according to the ZCW algorithm and 11 γ_PR_ angles. The spin models consist of a specific H spin and its neighboring H spins within the distance of 4.5 Å, corresponding to the four candidate structures of His1-4. The isotropic chemical shifts were taken from the experimental values, and the effective dipolar coupling for a specific spin pair H_j_ and H_k_ is considered:

(1) $$b_{\mathrm{rss},j}=\sqrt{\underset{j\neq k}{\sum }b_{\mathrm{jk}}^{2}}$$

where *b*_jk_ is the dipolar coupling between spins H_j_ and H_k_. The *J*-couplings and the chemical shift anisotropies of ^1^Hs were neglected. The other conditions are identical to the experiments.

### 2.5. Quantum Chemical (Density Functional Theory (DFT) and GIPAW) Calculation

The coordinates of all atoms were optimized (calculation mode ‘relax’ of program ‘pw’) before chemical shift calculation (program ‘gipaw’) using the density functional theory programs in the Quantum ESPRESSO package version 6.1 [58,59]. The cif2cell program was used to convert atomic coordinates from CIF to the unit cell. The unit cell contained 40 atoms for monoclinic l-histidine. The Monkhorst–Pack *k*-point grid was generated using a resolution of 0.4 Å^−1^. X.pbetrm-new-gipaw-dc. UPF was used as the pseudopotential for the atom X [60]. The kinetic energy cutoff for the wave functions (ecutwfc) was set to 80 Ry. The coordinates of all atoms were optimized with relaxed cell parameters starting from those determined by microED. The NMR shieldings, and thus chemical shifts, were obtained by subtracting the calculated chemical shifts from the reference shifts, which were adjusted to give the best agreement with the experimental data.

## 3. Results and Discussion

### 3.1. Ambiguities in MicroED Solution

**Figure 1:**
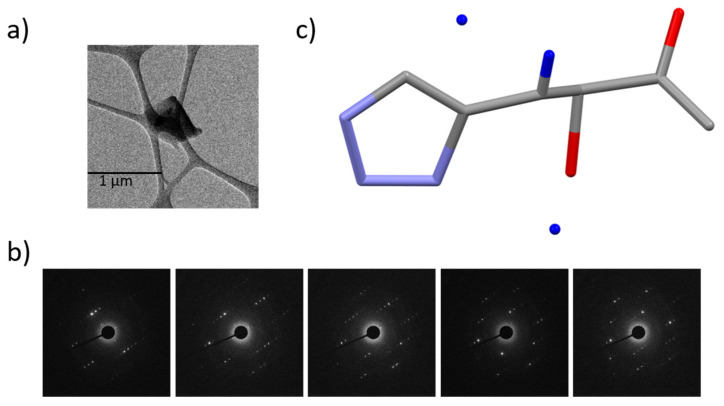
(**a**) TEM image of a single crystal of monoclinic l-histidine. (**b**) A set of ED patterns observed under the continuous rotation. The sample rotated 1° during each acquisition. (**c**) Initial structure solved using microED data set. The dark blue, grey, light blue, and red atoms denote H, C, N, and O, respectively.

**Figure 2:**
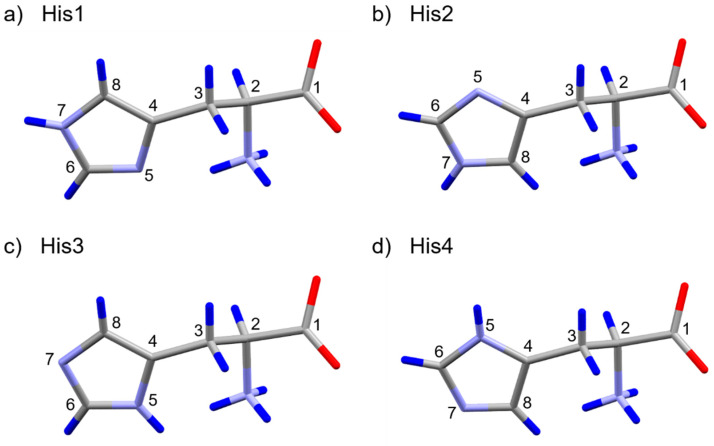
Four candidate structures of monoclinic l-histidine (His) derived from microED analysis: (**a**) His1, (**b**) His2, (**c**) His3, and (**d**) His4. The dark blue, grey, light blue, and red atoms denote H, C, N, and O, respectively. His1(3) differ from His2(4) by the positions of C and N atoms on the imidazole ring. His1(2) differ from His3(4) by the position of H atom on a N atom on the imidazole ring. The H, C and N atoms are numbered.

Figure 1a,b represent the TEM image of a monoclinic His single crystal and a part of its ED patterns used in the structural solution, respectively. The initial solution is obtained from a single data set from a single crystal without merging (Figure 1c). The data provide the lattice parameters of a = 5.18 Å, b = 7.37 Å, c = 9.45 Å, and β = 98.4° with a space group of monoclinic P2_1_ (Table S1). Although the initial structure includes missing or poorly located H(s) and misassignments of C, N, and O atoms, they are easily corrected with chemical knowledge of the molecular structure. In addition, a simple ^1^H/^14^N correlation experiment reveals the protonation states of the imidazole nitrogens and formation of zwitterion similar to the previous demonstration on orthorhombic l-histidine (see Figure S2) [4,43]. Since only two NH correlations appear and one is from NH_3_^+^ based on the characteristic ^14^N shifts, we confirm: (i) only one nitrogen on the imidazole ring was protonated and (ii) zwitterion was formed. However, there still remain four possible candidate structures derived from microED, which gave relatively high and indistinguishable R factors between 17.0 to 18.3 (Figure 2 and Table S2). We classify these structures into two groups—one consists of His1 (Figure 2a) and His3 (Figure 2c) while the other consists of His2 (Figure 2b) and His4 (Figure 2d). These two groups differ by the positions of C and N atoms on the imidazole ring. The R factors may indicate which is the correct structure; however, the kinetic approach in microED introduced ambiguities to distinguish these two atoms. Within each group, the His1 differs from His3 (as well as His2 differs from His4) by the position of H on either N5 or N7. Again the uncertainty in the R factor evaluation hampered clear validation of H atoms. In the folllowing, we used ^1^H-^14^N and ^1^H-^1^H NMR dipolar-based experiments to identify the correct structure.

### 3.2. Spectral Assignments

**Figure 3:**
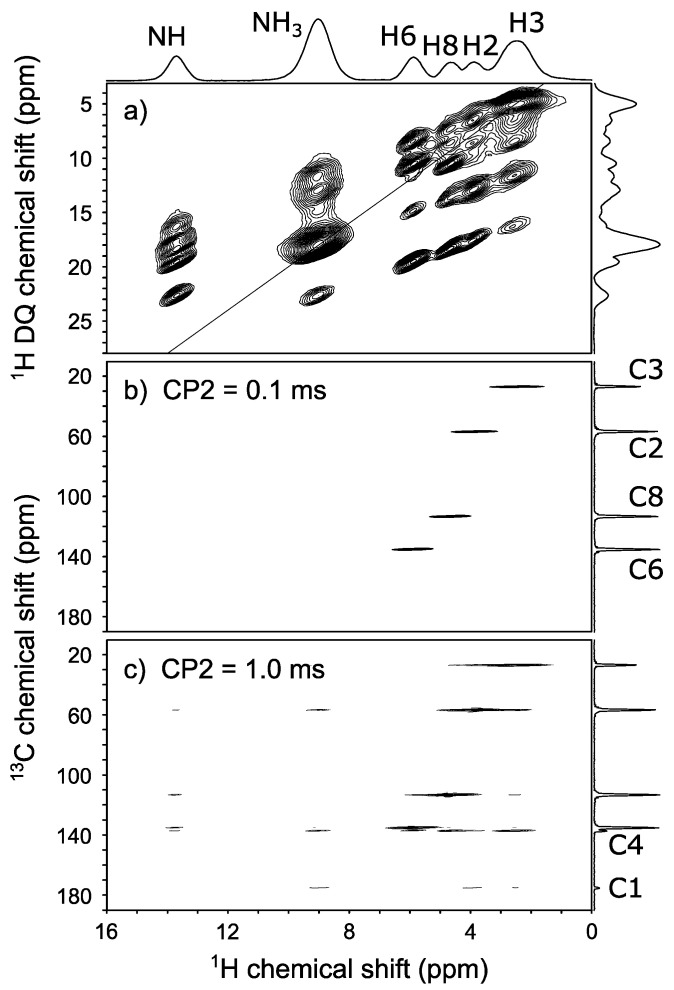
^1^H and ^13^C spectral assignments of His with (**a**) 2D ^1^H-^1^H $R18_{4}^{7}\;$experiment, (**b**) and (**c**) 2D ^1^H-^13^C proton-detected CP-HSQC experiments with (**b**) CP2 = 0.1 ms and (**c**) 1.0 ms.

In the NMR dipolar-based validation, time evolution of a specific ^1^H resonance under ^1^H-^14^N or ^1^H-^1^H dipolar couplings is simulated based on the candidate structures and compared with the experimental one. Thus, it is crucial to assign the ^1^H resonances correctly to each hydrogen prior to the ^1^H-^14^N PM-S-RESPDOR and ^1^H-^1^H SERP analyses. For that purpose, ^1^H DQ/^1^H SQ homonuclear and ^1^H/^13^C heteronuclear correlation experiments were performed and their spectra are shown in Figure 3. Commonly, the combination of ^1^H DQ/^1^H SQ correlations (Figure 3a) and their distances from the crystallographic structure is a powerful tool for assigning ^1^H resonances. However, the location of a hydrogen atom on the imidazole ring is uncertain, which can lead to errors in assignment if based on ^1^H-^1^H distances. Therefore, it is better to assign peaks by ^1^H/^13^C correlation experiments. With such short CP2 of 0.1 ms, Figure 3b only shows the covalently bonded ^1^H and ^13^C resonances. Based on ^13^C chemical shifts, we unambiguously assign the four peaks C3, C2, C8, and C6 and link them to the corresponding ^1^H resonances of H3, H2, H8, and H6. The two remaining ^1^H resonances that do not show the correlations in Figure 3b are clearly assigned to imidazole N**H** and N**H_3_** resonances since they do not attach to ^13^C. Based on the ^1^H signal intensities, we also assign N**H** and N**H_3_** with certainty. The ^1^H/^14^N correlation experiment (Figure S2) also supports this assignment. All ^1^H resonances are clearly assigned. Quaternary carbons can be assigned through long C–H correlations. We performed the CP-HSQC experiment with a longer CP2 of 1.0 ms. The spectrum in Figure 3c shows the two additional ^13^C peaks with different chemical shifts that we can clearly assign to C4 and C1 resonances.

#### ^1^H-^14^N PM-S-RESPDOR Experiment

Since the difference between His1(3) and His2(4) lies on the position of two N atoms on the imidazole ring, their H–N dipolar coupling network also differs. In order to probe the H–N dipolar coupling network, we performed the ^1^H-^14^N PM-S-RESPDOR experiment using the sequence in Figure S1c for the fraction curve. The fraction curve is generated by setting the ratio: Δ*S*/*S*_0_ = (*S*_0_ − *S*’)/*S*_0_ as a function of recoupling mixing time (*τ*_mix_), where *S*_0_ and *S*’ are obtained from PM-S-RESPDOR without and with a ^14^N saturation pulse, respectively. The intensity Δ*S*/*S*_0_ of the fraction curve could in principle strongly depend on the ^14^N chemical shifts, ^14^N quadrupolar coupling as well as the *ν*_14N_ of the saturation pulse. We overcame such dependences by using the PM pulse as the ^14^N saturation pulse since it can completely saturate ^14^N atoms no matter of ^14^N parameters [37]. ^1^H-^14^N PM-S-RESPDOR is capable of monitoring the weak ^1^H-^14^N dipolar couplings (i.e., long range ^1^H-^14^N interaction), which is relevant for the structural validation, even in the presence of strong ^1^H-^14^N dipolar coupling. This great advantage is owing to the recoupled Hamiltonians which commute to each other. As a result, while the behavior of the fraction curve at a short *τ*_mix_ is dominated by the strongest ^1^H-^14^N coupling, weak couplings modulate the fraction curve at a long *τ*_mix_. Our strategy for correcting the N positions was to evaluate the agreement between the experimental and simulated ^1^H-^14^N PM-S-RESPDOR fraction curves. The better agreement corresponds to the more plausible structure.

**Figure 4:**
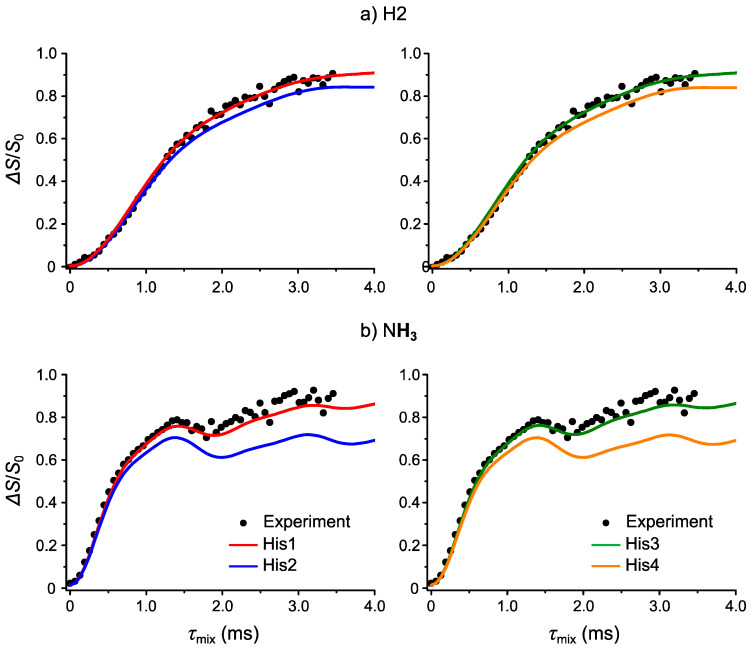
Comparison of the experimental ^1^H-^14^N phase-modulated rotational-echo saturation-pulse double-resonance (PM-S-RESPDOR) fraction curves (black circles) with the simulated curves of His1 (red), His2 (blue), His3 (green), His4 (orange) for (**a**) H2 and (**b**) NH_3_.

Figure 4a shows the experimental (black circles) ^1^H-^14^N PM-S-RESPDOR fraction curve of H2. We first notice that the Δ*S*/*S*_0_ of this fraction curve is larger than 0.67—a theoretical maximum for an isolated ^1^H-^14^N system, indicating that the fraction curve results from the contributions of more than one ^1^H-^14^N pair. In other words, H2 experiences the dephasing effect induced by not only the closest neighboring ^14^N atom but also multiples of surrounding ^14^N atoms. We then simulated the ^1^H-^14^N fraction curve of this H2 atom using the ^1^H-^14^N distances, and thus dipolar couplings taken from the four candidate structures. To run the simulations within a reasonable time, we set the cutoff ^1^H-^14^N distance of 4.0 Å, corresponding to a dipolar coupling constant of 0.136 kHz. Figure 4a shows that the simulated curves of His1 (red) and His3 (green) are in better agreement with the experiment (black circles) than those of His2 (blue) and His4 (orange). However, the differences between the simulated curves for all structures are not significant enough to identify whether His1(3) are favored. Hence, we next examined the NH_3_ site.

Figure 4b shows the experimental (black circles) ^1^H-^14^N fraction curve of NH_3_. By contrast with that of H2, this fraction curve first reaches a maximum before continuing to grow at longer mixing time. This maximum results from the dipolar coupling of the covalent H–N bond. Since microED cannot precisely determine H–N distance in this case, we should separately measure this N–H dipolar coupling for better reproduction of the experiment. This was fulfilled by adjusting the strongest H–N dipolar coupling in a two-spin ^1^H-^14^N simulation so that the *τ*_mix_ for the first maximum between simulation and experiment matches (see Figure S3). Another issue to consider is the fast rotation of three H atoms of the NH_3_ site along the C–N axis. In the NMR timescale, N-H dipolar couplings, regardless to bonded or non-bonded NH, are time averaged dipolar coupling rather than the summation of the three independent N–H dipolar couplings. Again, in order to reproduce the experiment, we need to derive this averaged N-H dipolar coupling, whose procedure is detailed in the SI and Figure S4. Once we had calculated the averaged dipolar couplings of covalently bonded and the non-bonded H–N(s), we then simulated the ^1^H-^14^N PM-S-RESPDOR fraction curve for the NH_3_ site of the four candidate structures. Figure 4b shows a significantly better agreement between the simulated fraction curves of His1 (red) and His3 (green) and the experiment than those of His2 (blue) and His4 (orange). This confirms that His1 and His3 are favored, allowing the clear assignments of C and N atoms. Besides H2 and NH_3_ sites, we also simulated the ^1^H-^14^N PM-S-RESPDOR fraction curves for other ^1^H atoms but the experimental and simulated curves from the four candidate structures are similar to each other; hence, we cannot reach any conclusion from these ^1^H atoms (Figure S5).

In conclusion, by performing ^1^H-^14^N S-RESPDOR in combination with the PM pulse, we can identify the plausible structures, thus assign the C and N atoms with certainty on the imidazole ring.

### 3.3. ^1^H-^1^H SERP Experiments

Thanks to the ^1^H-^14^N PM-S-RESPDOR experiment, the candidate structures from microED were limited to His1 and His3. These two structures differ by the H position on either N7 or N5 on the imidazole ring. For His1, this H is spatially close to both H6 and H8 (*d*_H-H_ ≤ 3.0 Å) whereas for His3, this H is only spatially close to H6. Such a difference in ^1^H-^1^H dipolar coupling network results in a different magnetization transfer profile. Namely, if we can selectively transfer the magnetization from NH to H6 and H8, we expect that His1 should provide two ^1^H-^1^H build-up curves with similar build-up rates whereas His3 should provide two ^1^H-^1^H build-up curves with different build-up rates. By simulating these curves and comparing them with the experiments, we can identify the correct structure. This strategy is feasible only when the magnetization transfer is selective otherwise the other ^1^H-^1^H dipolar couplings would complicate this transfer. For example, under a broadband recoupling sequence such as fp-RFDR, the build-up curves show almost similar build-up rates for all ^1^H atoms owing to the relayed magnetization transfer [40]. For this reason, we performed the ^1^H-^1^H SERP experiments to selectively transfer the magnetization between a ^1^H-^1^H pair, using the sequence in Figure S1d.

In the previous section, we measured the H–N bond distance by matching the simulated and experimental ^1^H-^14^N PM-S-RESPDOR curves because microED fails to precisely locate H atoms for covalent N–H bond. With the new N–H distance, the new H position must be modified. Consequently, the distances of this N-bonded H to other H atoms also need to be recalculated for reproducing the experiment. The procedure of recalculation is shown in the SI and Figure S6. For running simulations within a reasonable time, we set the cutoff ^1^H-^1^H distance of 4.5 Å, corresponding to a dipolar coupling constant of 1.32 kHz.

**Figure 5:**
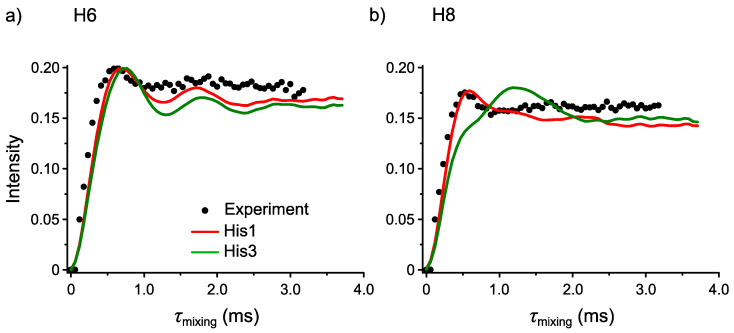
Comparison of the experimental ^1^H-^1^H selective recoupling of proton (SERP) build-up curve (black circle) with the simulated curves from His1 (red) and His3 (green) for selective transfer between (**a**) NH-H6 and (**b**) NH-H8.

Figure 5a shows the comparison of the experimental ^1^H-^1^H SERP build-up curve with the simulated curves from His1 (red) and His3 (green) for selective transfer between NH and H6. All curves are similar to each other. This result is expected because the NH is spatially close to H6 for both His1 and His3; thus, their build-up curves should be similar. Figure 5b shows the comparison of the experimental ^1^H-^1^H SERP build-up curve with the simulated curves from His1 (red) and His3 (green) for selective transfer between NH and H8. We clearly observe that the simulated build-up curve from His1 matches the experimental curve well but the curve from His3 does not. Furthermore, the simulated NH-H8 build-up rate is similar to that of NH-H6 for His1. These results clearly indicate that His1 is the correct structure.

We note that the ambiguity of the H position here can be qualitatively solved by the ^1^H-^1^H $R12_{2}^{5}$ sequence (Figure S1a) at short mixing time of 57.1 μs, where only short *d*_H-H_ correlations can be probed. The experiment clearly gives two correlations between NH and H6/H8 with similar intensities, confirming that His1 is the correct structure (see Figure S7). This is consistent with the result from SERP.

In conclusion, the ^1^H-^1^H SERP experiments reveal the H position on the N7 atom. 

We have further performed quantum chemical calculation based on a well-established NMR crystallography approach. First, geometry optimization was conducted by relaxing all atoms for His1-4. The root mean square of the atomic displacement of non-hydrogen atoms is given in Table S3. The large atomic displacements in His2-4 reveal that these structures are unstable and need significant structural modifications to minimize the energy. On the other hand, His1 clearly exhibits the minimum atomic displacement, indicating His1 is the most plausible structure. The His1 structure also exhibits the minimum energy consistent with all the above analyses (Table S3). The ^1^H/^13^C/^15^N isotropic chemical shifts were calculated for each geometry optimized structure and compared with the experimental values (Figure S8 and Table S4). The best agreements in ^1^H and ^13^C shifts are given for the His1 structure. All the above analyses consistently support the assertion that His1 is the correct structure, agreeing with the published XRD monoclinic l-histidine structure from CCDC. This conclusion is further confirmed by the comparison of the two structures (see Figure S9).

## 4. Conclusions

We have demonstrated that the ssNMR dipolar-based experiments can provide a reliable measure of structure validation. While microED successfully solves the structures of sub-micro sized crystals, several different candidates remain due to uncertain H positions and C/N/O assignments. R factor is widely used as a reliable measure of validation in XRD; however, the nature of dynamic scattering in microED undermines its reliability in kinetic analysis. On the other hand, it is experimentally and numerically feasible to investigate the spin evolution for each candidate structure in the ssNMR dipolar-based method to identify the correct structure. As a demonstration, the crystalline structure of monoclinic l-histidine was solved. MicroED provided the four candidate structures each of which differs in H positions and C/N assignments. ssNMR dipolar-based experiments were used with the aid of spin dynamics numerical simulations for structural validation. Namely, by comparing the experimental and simulated data, we unambiguously distinguished the C and N atoms with ^1^H-^14^N PM-S-RESPDOR and correctly located the H atom on the N atom of the imidazole ring with ^1^H-^1^H SERP. The chemical shifts, atomic displacement during geometry optimization, and total energy in the quantum chemical calculations also supported the result. The ssNMR dipolar-based experiments do not need any isotopic labelling and were performed at natural abundance with high sensitivity of ^1^H-detection. The additional advantage of our approach is the independence from the temperature effect since all the experimental and simulated measurements were performed at the ambient temperature. Nevertheless, we note that the unambiguous ^1^H assignments are a prerequisite for the success of this approach because the ssNMR dipolar-based experiments studied the time evolution of each single ^1^H resonance. This requirement may be difficult for large molecules without isotopic labelling where the ^1^H resonances are severely overlapped. With the advantages and disadvantages, we believe that our approach still contributes to the NMR crystallography field for the structural determination of small molecules.

## Data Availability

Data available upon request.

## Supplementary Materials

The following are available online, Table S1: Crystallographic data for monoclinic l-histidine, Table S2: R factors for His1-4, Table S3: Root mean square of atomic displacement during energy optimization and total energies for His1-4, Table S4: The root mean square deviation value between experimental and calculated chemical shifts for His1-4, Figure S1: Pulse sequences for (a) 2D ^1^H DQ/^1^H SQ, (b) 2D ^1^H-{^13^C} CP-HSQC, (c) ^1^H-^14^N PM-S-RESPDOR, (d) ^1^H-^1^H SERP, and (e) 2D ^1^H-{^14^N} T-HMQC. For (a), $R18_{4}^{7}$ or $R12_{2}^{5}$ are used to excite and reconvert the ^1^H DQ coherences. For (c), the PM pulse is used to uniformly saturate the populations of ^14^N nuclei. For (d), the Q3 Gaussian pulse is used to selectively invert a ^1^H site of interest and *m* denotes the number of loops. The ^1^H-^14^N dipolar coupling is recovered by (b) $\mathrm{SR}4_{1}^{2}$ lasting for *τ*_mix_ on ^1^H channel or (e) long continuous wave pulse lasting for *τ*_p_ on ^14^N channel. The grey rand black rectangles denote the π/2 and π pulses, respectively, Figure S2: The 2D ^1^H-{^14^N} T-HMQC experiment using the sequence in Figure S1e was performed at *B*_0_ = 16.4 T and *ν*_R_ = 62.5 kHz. The *τ*_p_ was 350 µs while the ^1^H pulse lengths for π/2 and π pulses were 0.93 and 2.13 µs, respectively. The ^1^H and ^14^N offsets were 8 and −150 ppm, respectively. The 2D spectrum was recorded with 8 scans, 32 *t*_1_ points, and rotor-synchronized *t*_1_ increment of 16 µs. The recycling delay was 10 s. The experimental time was 1.4 h. The States-TPPI method was employed for the quadrature detection along the indirect dimension, Figure S3: ^1^H-^14^N PM-S-RESPDOR fraction curves: experiment (black circles) and ^1^H-^14^N simulation (red line) for (a) N**H_3_** and (b) N**H**. The ^1^H-^14^N dipolar coupling is numerically adjusted so that the *τ*_mix_ for the first maximum (dashed line) between experiment and simulation matches to each other, Figure S4: Schematic illustrations of calculating the temporal H position (H_temp_) resulting from the rotation of three hydrogens H_a_, H_b_, and H_c_ in the (a) three-dimensional and (b) simplified two-dimensional space. The $\vec{\mathrm{CN}}$ vector crosses the plane (H_a_H_b_H_c_) at the point R. The $\vec{{\mathrm{RH}}_{\mathrm{cross}}}$ and $\vec{{\mathrm{RH}}_{\mathrm{temp}}}$ vectors are defined by the mathematical at the upper right of the figure. The symbol *α*(*t*) denotes an angle as a variable of time between $\vec{{\mathrm{RH}}_{a}}$ and $\vec{{\mathrm{RH}}_{\mathrm{temp}}}$ vectors, Figure S5: Comparison of the experimental ^1^H-^14^N RESPDOR fraction curve (black circles) with the simulated curves from His1 (red), His2 (blue) for (a) N**H**, (b) H6, and (c) H3. For a, the dipolar coupling of the H–N bond was taken from the two-spin simulation in Figure S3b, Figure S6: Schematic illustration to re-calculate the ^1^H-^1^H distance of interest, here the H_new_−H_far_ pair based on the new *d*_N-Hnew_ derived from the ^1^H-^14^N PM-S-RESPDOR experiment, Figure S7: The 2D ^1^H DQ/^1^H SQ $R12_{2}^{5}$ experiment using the sequence in Figure S1a was performed at *B*_0_ = 16.4 T and ν_R_ = 70 kHz. The $R12_{2}^{5}$ DQ homonulcear recoupling sequence was used to excite and reconvert the ^1^H DQ coherences. The N**H**-H6 and N**H**-H8 correaltions are highlighted by two horizontal lines. The ν_1H_ was 210 kHz for $R12_{2}^{5}\;$and 281 kHz for the read-pulse of 0.89 µs. The $R12_{2}^{5}\;$mixing time was 57.1 µs. Before the read-pulse, the 0.5 ms z-filter was employed to suppress residual transversal magnetization. The 2D ^1^H DQ/SQ spectrum was recorded with four scans, 64 *t*_1_ points, and rotor-synchronized *t*_1_ increment of 57.1 µs. The recycling delay was 10 s. The experimental time was 1.4 h. The States-TPPI method was employed for the quadrature detection along the indirect dimension, Figure S8: A comparison of the experimental chemical shifts with the calculated values by GIPAW for His1 (black squares), His2 (red circles), His3 (blue triangles), and His4 (green crosses) for (a) ^1^H, (b) ^13^C, and (c) ^15^N, Figure S9: Comparison of the (a) His1 structure with (b) the XRD-based structure, SIMPSON input files for PM-S-RESPDOR and SERP.

## Sample Availability

Sample of the monoclinic form of l-histidine compound is available from the authors.
