# Supplementary material for: Structure Solution of Nano-Crystalline Small Molecules Using MicroED and Solid-State NMR Dipolar-Based Experiments
Source: Molecules. 2021 Jul 31;26(15):4652. doi: 10.3390/molecules26154652 (PMC8347922; doi:10.3390/molecules26154652)
Supplement: Supplementary file 1 [file molecules-26-04652-s001.zip › Supplementary_material_submitted.pdf]

Supplementary material

Table S1. Crystallographic data for monoclinic L-histidine

Camera length [mm]	874
Δϕ [°/frame]	1
Number of frames	60
ϕ range [°]	-30 to +30
Space group	$P2_1$
Unit cell axes [Å]	
a	5.18
b	7.37
c	9.45
β[°]	98.43
Z	2
V [$Å^3$]	357
Resolution [Å]	5.79 – 0.70
Number of independent reflections	589
Completeness [%]	49.8
$I/\sigma(I)$	4.39
$CC_{1/2}$[%]	99
R_{meas}[%]	13.7

Table S2. R factors for His1-4

	His1	His2	His3	His4
R factor [%]	17.06	17.82	17.51	18.33

Table S3. Root mean square of atomic displacement during energy optimization and total energies for His1-4

	His1	His2	His3	His4
Root mean square of atomic displacement [Å]	0.062	0.513	1.609	0.664
Energy [Ry]	-405.42	-405.37	-405.36	-405.38

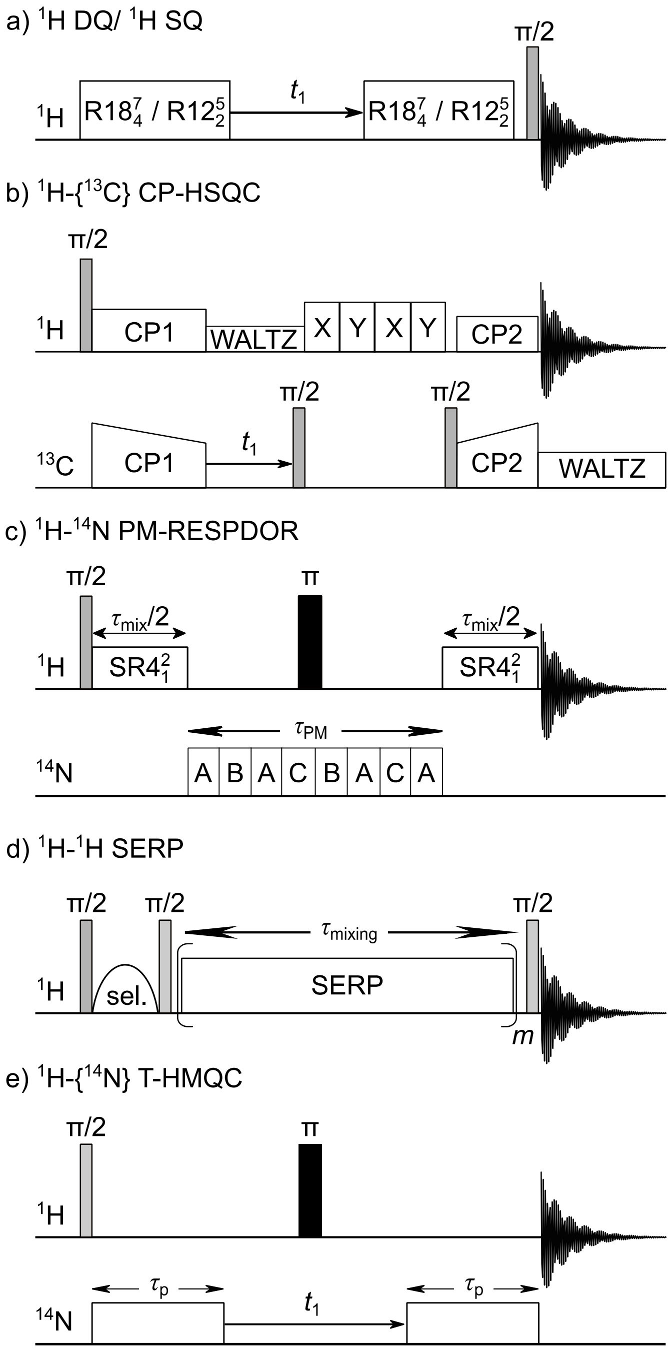

Figure S1. Pulse sequences for (a) 2D ^{1}H DQ/ ^{1}H SQ, (b) 2D ^{1}H-{^{13}C} CP-HSQC, (c) ^{1}H-^{14}N PM-RESPDOR, (d) ^{1}H-^{1}H SERP, and (e) 2D ^{1}H-{^{14}N} T-HMQC. For (a), $\mathrm{R}18_4^7$ or $\mathrm{R}12_2^5$ are used to excite and reconvert the ^{1}H DQ coherences. For (c), the PM pulse is used to uniformly saturate the populations of ^{14}N nuclei. For (d), the Q3 Gaussian pulse is used to selectively invert a ^{1}H site of interest and *m* denotes the number of loop. The ^{1}H-^{14}N dipolar coupling is recovered by (b) $\mathrm{SR}4_1^2$ lasting for τ_{mix} on ^{1}H channel or (e) long continuous wave pulse lasting for τ_{p} on ^{14}N channel. The grey rand black rectangles denote the π/2 and π pulses, respectively.

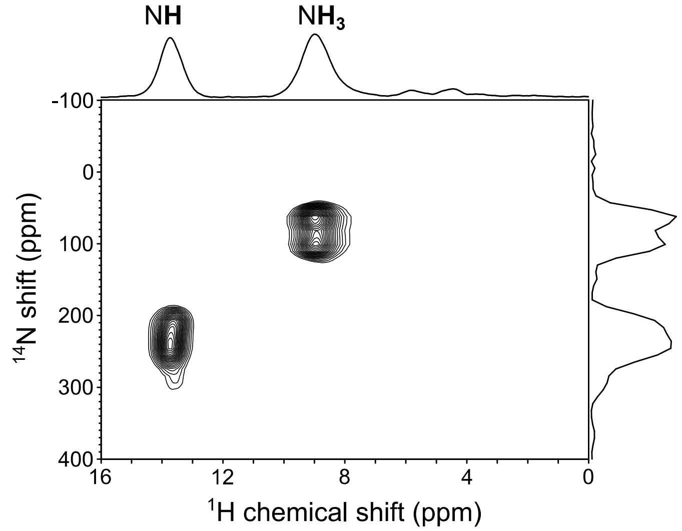

Figure S2. The 2D ^{1}H-{^{14}N} T-HMQC experiment using the sequence in Figure S1e was performed at B_0 = 16.4 T and ν_R = 62.5 kHz. The τ_p was 350 µs while the ^{1}H pulse lengths for π/2 and π pulses were 0.93 and 2.13 µs, respectively. The ^{1}H and ^{14}N offsets were 8 and -150 ppm, respectively. The 2D spectrum was recorded with 8 scans, 32 t_1 points, and rotor-synchronized t_1 increment of 16 µs. The recycling delay was 10 s. The experimental time was 1.4 hours. The States-TPPI method was employed for the quadrature detection along the indirect dimension.

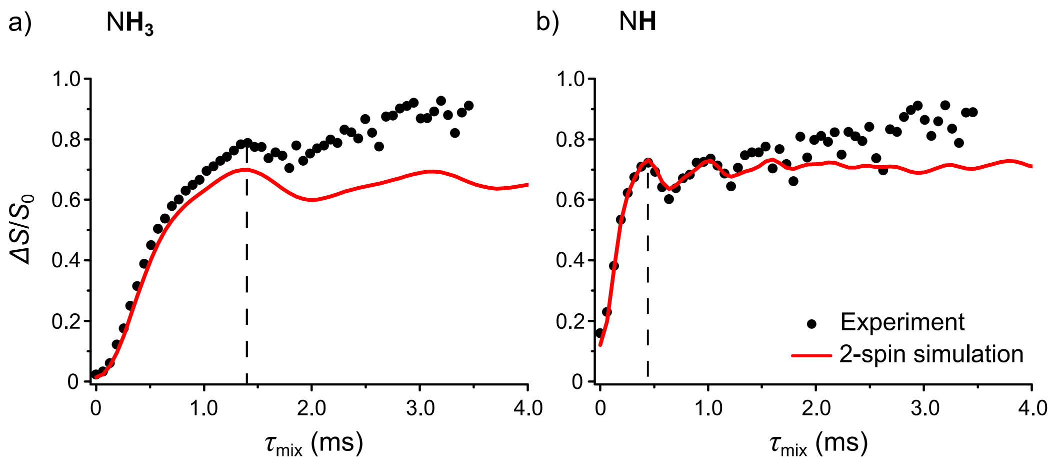

Figure S3. ^{1}H-^{14}N PM-RESPDOR fraction curves: experiment (black circles) and ^{1}H-^{14}N simulation (red line) for a) N**H₃** and b) N**H**. The ^{1}H-^{14}N dipolar coupling is numerically adjusted so that the τ_{mix} for the first maximum (dashed line) between experiment and simulation matches to each other.

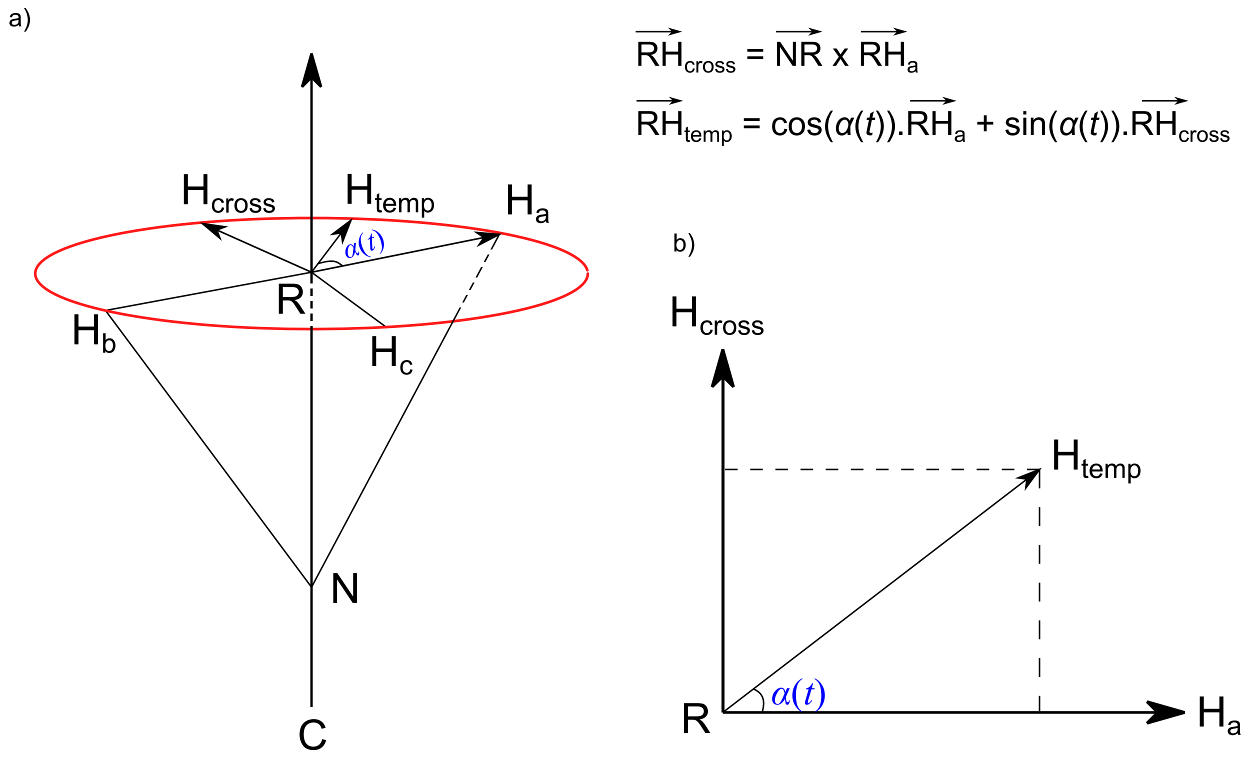

Figure S4. Schematic illustrations of calculating the temporal H position (H_{temp}) resulting from the rotation of three hydrogens H_a, H_b, and H_c in the a) three-dimensional and b) simplified two-dimensional space. The $\overrightarrow{\mathrm{CN}}$ vector crosses the plane ($H_aH_bH_c$) at the point R. The $\overrightarrow{\mathrm{RH_{cross}}}$ and $\overrightarrow{\mathrm{RH_{temp}}}$ vectors are defined by the mathematical at the upper right of the figure. The symbol $\alpha(t)$ denotes an angle as a variable of time between $\overrightarrow{\mathrm{RH_a}}$ and $\overrightarrow{\mathrm{RH_{temp}}}$ vectors.

From the microED-derived candidate structures, we can extract the C-NH_3 indices. We denote the three H atoms as H_a, H_b, and H_c. To determine the averaged H-N dipolar coupling, we follow these steps:

1. Determining the point R:

$$\overrightarrow{\mathrm{CN}} \cap (\mathrm{H_a H_b H_c}) = \{\mathrm{R}\} \tag{S1}$$

2. Determining the vector $\overrightarrow{\mathrm{RH_{cross}}}$:

$$\overrightarrow{\mathrm{RH_{cross}}} = \overrightarrow{\mathrm{NR}} \times \overrightarrow{\mathrm{RH_a}} \tag{S2}$$

3. Determining the point H_{temp}:

$$\overrightarrow{\mathrm{RH_{temp}}} = \cos(\alpha(t))\,\overrightarrow{\mathrm{RH_a}} + \sin(\alpha(t))\,\overrightarrow{\mathrm{RH_{cross}}} \tag{S3}$$

, where $\alpha(t)$ is an angle between $\overrightarrow{\mathrm{RH_a}}$ and $\overrightarrow{\mathrm{RH_{ave}}}$ vectors, ranging from 0 to 2π. From the $\overrightarrow{\mathrm{RH_{ave}}}$ vector, we can calculate the indices of H_{temp}, which is a function of the angle $\alpha(t)$.

4. Determining the averaged H-N dipolar coupling:

$$\overline{b_{H-N}} = \frac{1}{2\pi}\int_0^{2\pi} \frac{120.1}{(d_{H_{temp}-N})^3} . \frac{\gamma_{14N}}{\gamma_{1H}}\, d\alpha \tag{S4}$$

Note: this procedure only applies for non-bonded N atoms. For the covalently bonded N atom, the H-N dipolar coupling should be determined by simulations as shown in Figure S3.

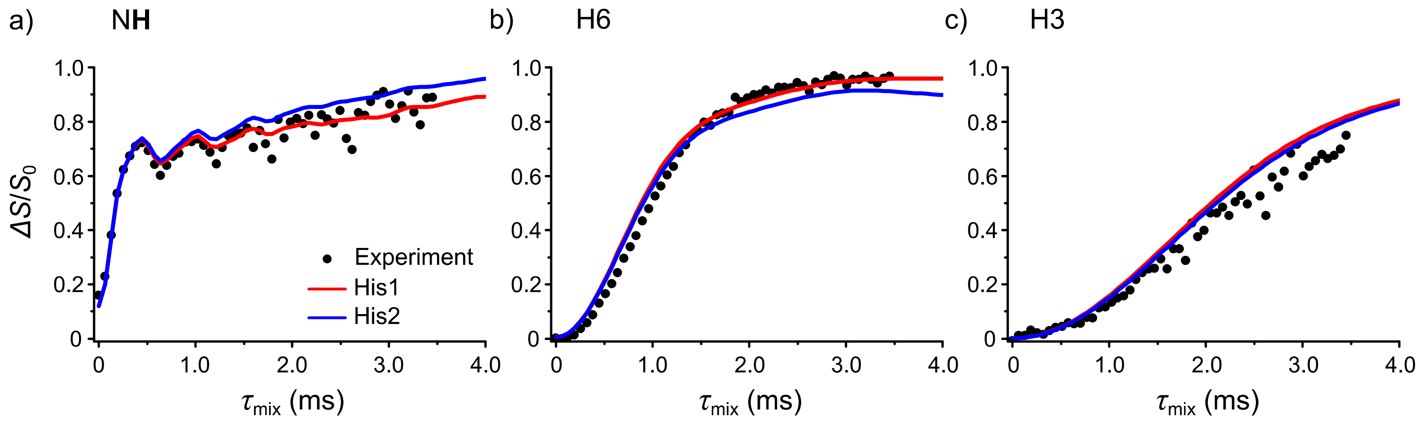

Figure S5. Comparison of the experimental ^{1}H-^{14}N RESPDOR fraction curve (black circles) with the simulated curves from His1 (red), His2 (blue) for a) N**H**, b) H6, and c) H3. For a, the dipolar coupling of covalent H–N bond was taken from the two-spin simulation in Figure S3b.

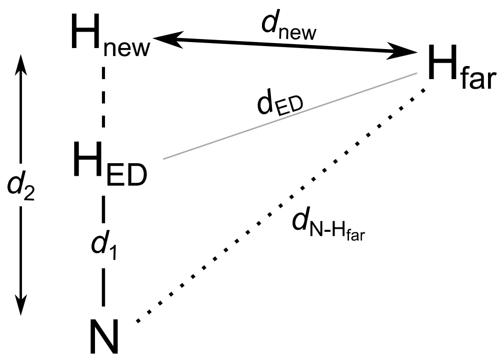

Figure S6. The schematic illustration to re-calculate the ^{1}H-^{1}H distance of interest, here H_{new}-H_{far} pair based on the new $d_{N\text{-}Hnew}$ derived from ^{1}H-^{14}N PM-RESPDOR experiment.

From the N–H_{new} distance taken from ^{1}H-^{14}N PM-RESPDOR experiment, we can locate the position of H_{new}. Hence, the H_{new}–H_{far} distance needs to be recalculated for the input of SERP simulations. As $^{1}H_{ED}$, $^{1}H_{new}$, N, and H_{far}, are in the same plane, we can apply an equation relating the sides of a triangle with an angle:

$$\frac{(d_1)^2 + (d_{N-H_{far}})^2 - (d_{ED})^2}{2.\,d_1.\,d_{N-H_{far}}} = \frac{(d_2)^2 + (d_{N-H_{far}})^2 - (d_{new})^2}{2.\,d_2.\,d_{N-H_{far}}} \tag{S5}$$

Simplifying and rearranging the equation, we can yield the refined $r_{1H\text{-}35Cl}$:

$$\frac{(d_1)^2 + (d_{N-H_{far}})^2 - (d_{ED})^2}{d_1} = \frac{(d_2)^2 + (d_{N-H_{far}})^2 - (d_{new})^2}{d_2} \tag{S6}$$

$$\Rightarrow\ d_{new} = \sqrt{(d_2)^2 + (d_{N-H_{far}})^2 - \frac{d_2}{d_1}.\left[(d_1)^2 + (d_{N-H_{far}})^2 - (d_{ED})^2\right]} \tag{S7}$$

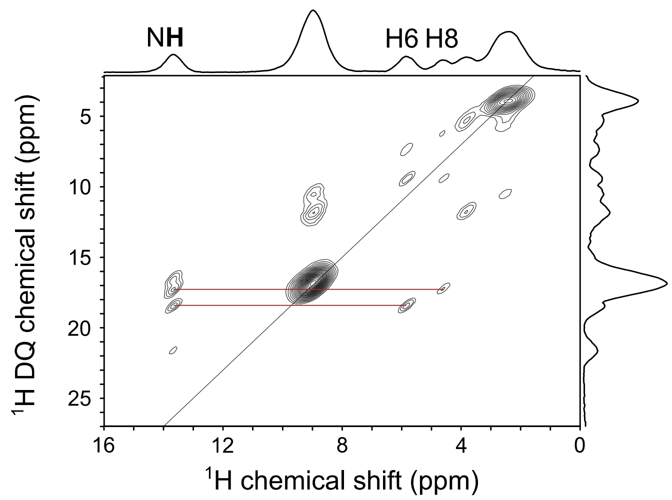

Figure S7. The 2D ^{1}H DQ/ ^{1}H SQ $\mathrm{R}12_2^5$ experiment using the sequence in Figure S1a was performed at B_0 = 16.4 T and ν_R = 70 kHz. The $\mathrm{R}12_2^5$ DQ homonulcear recoupling sequence was used to excite and reconvert the ^{1}H DQ coherences. The N**H**-H6 and N**H**-H8 correaltions are highlighted by two horizontal lines. The ν_{1H} was 210 kHz for $\mathrm{R}12_2^5$ and 281 kHz for the read-pulse of 0.89 µs. The $\mathrm{R}12_2^5$ mixing time was 57.1 µs. Before the read-pulse, the 0.5 ms z-filter was employed to suppress residual transversal magnetization. The 2D ^{1}H DQ/SQ spectrum was recorded with 4 scans, 64 t_1 points, and rotor-synchronized t_1 increment of 57.1 µs. The recycling delay was 10 s. The experimental time was 1.4 hours. The States-TPPI method was employed for the quadrature detection along the indirect dimension.

Cross-verification with quantum chemical calculation

We additionally performed GIPAW chemical shift calculation for ^{1}H, ^{13}C, and ^{15}N (cal. δ_{iso}) for His1-4. Figure S8 shows the comparison of the ^{1}H (a), ^{13}C (b), and ^{15}N (c) experimental (exp. δ_{iso}) and calculated (cal. δ_{iso}) chemical shifts for His1-4.

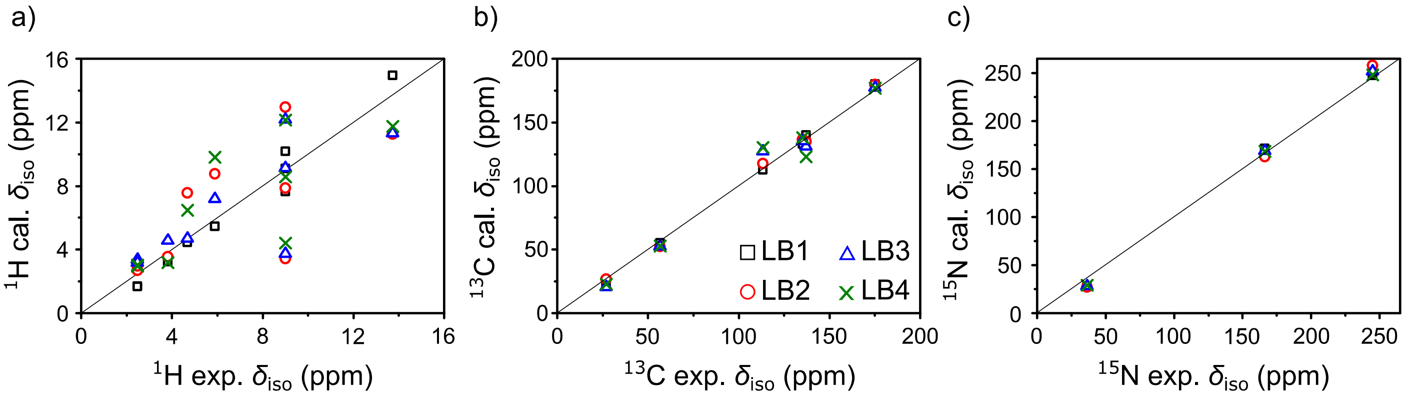

Figure S8. Comparison of the experimental chemical shifts with the calculated values by GIPAW for His1 (black squares), His2 (red circles), His3 (blue triangles), and His4 (green crosses) for a) ^{1}H, b) ^{13}C, and c) ^{15}N.

Table S4. The root mean square deviation value between experimental and calculated chemical shifts for His1-4

	^{1}H (ppm)	^{13}C (ppm)	^{15}N (ppm)
His1	0.61	3.20	6.22
His2	1.68	3.26	9.46
His3	1.51	7.03	6.48
His4	1.69	9.46	4.77

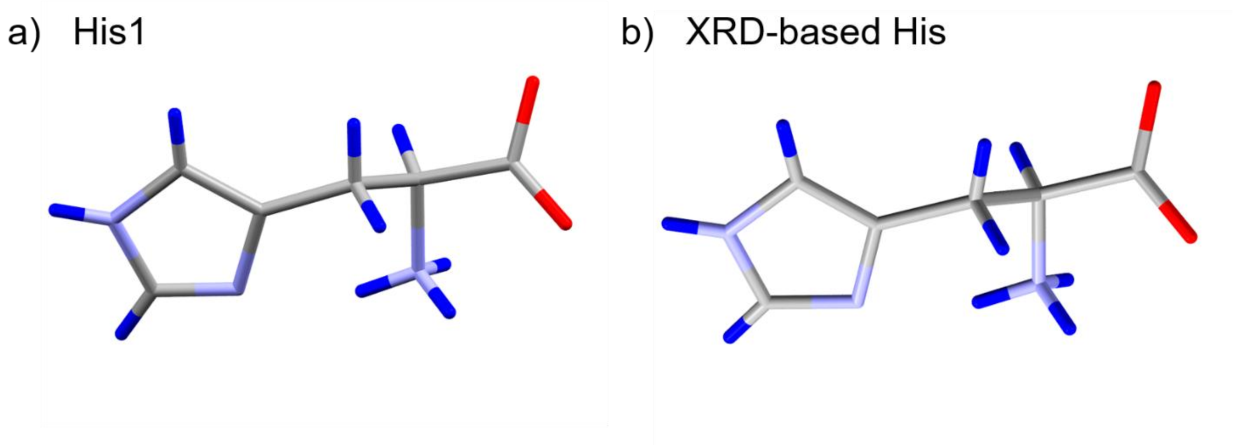

Figure S9. Comparison of the a) His1 structure with b) the XRD-based structure.
